# Editorial: Functional food ingredients and intestinal homeostasis

**DOI:** 10.3389/fnut.2023.1243424

**Published:** 2023-07-07

**Authors:** Bo Li, Yi Charlie Chen

**Affiliations:** ^1^Department of Tea Science, Zhejiang University, Hangzhou, China; ^2^College of Health, Science, Technology and Mathematics, Alderson Broaddus University, Philippi, WV, United States

**Keywords:** functional food ingredient, intestinal homeostasis, intestinal immunity, intestinal barrier, gut microbiota

The intestine is not only an organ for absorption and digestion, but also plays an important role in immunity and incretion. Intestinal health relies on the establishment of homeostasis, a dynamic equilibrium state formed by the interaction of the host (such as intestinal mucosa, and immune barrier) and the intestinal environment including gut microbiota, nutrients and metabolites. The mucosal barrier mainly consists of intestinal epithelial cells (IECs) that are linked by tight junctions (TJs) and the protective mucous layer. The different types of IECs originally differentiate from intestinal stem cells (ISCs), and include the absorptive enterocytes and various types of secretory cells (Paneth cells that secrete antibiotics, tuff cells, goblet cells that secrete mucus, and enteroendocrine cells that secrete hormones). The mucous layer is above the epithelial barrier, and mainly contains the microbiota, secretory IgA, mucins, antimicrobial peptides and glycocalyx. Defects in intestinal barrier integrity result in increased intestinal permeability and development of intestinal inflammation and inflammatory bowel disease (IBD). The intestinal immune system contains the lamina propria (LP) and gut-associated lymphoid tissues (GALT), such as mesenteric lymph nodes and Peyer's patches (PP). Macrophages, B cells, T cells, M cells, dendritic cells (DCs) and natural killer cells are important components (1, 2). More than 100 trillion bacteria inhabit the lower gastrointestinal tract of humans, and their collective genome (the “microbiome”) contains at least 100 times as many genes as our own genome. Gut microbiota are critical in shaping host immunity, and have been considered as “forgotten organ” due to its closed association with human health. The imbalance of intestinal flora is associated with the occurrence of various diseases, including IBD, colorectal cancer, obesity, diabetes, cardiovascular diseases, alcoholic and nonalcoholic fatty liver disease, neurological and psychiatric disorders, etc. (3).

Various functional food ingredients and nutrients have been found to benefit intestinal health through promoting intestinal epithelial renewal and barrier function, restoring intestinal flora, inhibiting intestinal inflammation and tumor growth, regulating nutrient transport and incretin secretion. On the one hand, these ingredients maintain the intestinal homeostasis and human health by interacting intestinal epithelium and affecting the population structure, metabolism, and function of gut microbiota. On the other hand, the gut microbiota is involved in the bioconversion of natural active components, such as demethylation, deprenylation, deglycosylation, dihydroxylation and acetylation, thereby influencing their metabolism and biological activity (4).

This Research Topic is aimed at collecting papers studying functional components of foods that maintain intestinal homeostasis and improve related health problems and diseases. All papers in this special e-collection cover the above-mentioned aspects.

Data from Medina-Larqué et al. show that consumption of cranberry polyphenols (CP) and agavins (AG) regulate gut microbiota, barrier function, mucosal immunity and glucose metabolism in C57BL6 male mice fed an obesogenic high-fat and high-sucrose (HFHS) diet. CP increases the relative abundance of *Akkermansia muciniphila*, an anti-obesity gut symbiont. AG, either alone or combined with CP, promotes the glycan-degrading bacteria *Muribaculum intestinale, Faecalibaculum rodentium, Bacteroides uniformis*, and *Bacteroides acidifaciens*. CP and AG, alone or combined, increase toll-like receptor (TLR)-2 (Tlr2) expression and decrease interleukin 1ß (ILß1) expression. AG selectively upregulates the anti-inflammatory marker forkhead box P3 (Foxp3), while CP enhances the expression of NOD-like receptor family pyrin domain containing 6 (Nlrp6) inflammasome. Data from Yu et al. show that resveratrol effectively alleviates the intestinal symptoms of celiac disease in Caco-2 cells and mouse model induced by wheat gluten. Resveratrol regulates autoimmunity via upregulating Aire and Ubd genes, promotes nutrient absorption in intestine by downregulating Fgf15 and Nr0b2 genes, and reduces intestinal oxidative stress and inflammatory damage through activating PPAR, AMPK and FoxO signaling pathways.

Environmental factors usually have a significant effect on the growth and intestinal homeostasis of organisms. Fan et al. assess the effect of dietary phosphorus supplementation on growth, intestinal antioxidant activity, physical barrier function, and microflora of Songpu mirror carp (*Cyprinus carpio* Songpu) at the high concentration carbonate alkalinity environment. Appropriate phosphorus supplement effectively increases the activities of intestinal antioxidant enzymes including glutathione peroxidase (GSHPx) and superoxide dismutase (SOD), and regulates the corresponding gene expression via the Keap1-Nrf2 signaling pathway. In addition, phosphorus promotes intestinal immunity by upregulating anti-inflammatory genes and downregulating pro-inflammatory genes, and increases beneficial microflora. Phosphorus supplementation may be an effective strategy to ameliorate chronic carbonate alkaline stress in fish.

Food allergy is a typical mast cell and IgE-mediated immune response that causes immediate hypersensitivity, while food sensitivity is an IgG-mediated immune response that causes delayed hypersensitivity. An association is known between IgE-mediated food allergy and increased intestinal permeability, but the relation between IgG-mediated food sensitivity and permeability has not been well-described. Vita et al. found that IgG-mediated food sensitivities were significantly and positively associated with the intestinal permeability, with increasing antibodies to lipopolysaccharide (LPS) and occlude in human.

In summary, the above-mentioned studies focused on small molecules, macromolecules and inorganic elements including polyphenols, fructans, immunoglobulins and phosphorus, and revealed their health benefits for intestinal homeostasis in mice, fish and human. These data expand our understanding of the effects of food and nutrients on intestinal function and related mechanisms. It is hoped that more functional food ingredients can be used as a potential alternative to prevent and improve complex diseases by regulating intestinal homeostasis.

## Author contributions

All authors listed have made a substantial, direct, and intellectual contribution to the work and approved it for publication.
